# Long-term Risks of Recurrent Febrile Seizures

**DOI:** 10.15844/pedneurbriefs-34-22

**Published:** 2020-12-18

**Authors:** Daniel A. Freedman, Jorge Vidaurre

**Affiliations:** 1 Department of Pediatrics and Neurology, Nationwide Children's Hospital, OH

**Keywords:** Prognosis, Febrile Seizures, Epilepsy, Psychiatric Diagnosis, Seizure Disorders

## Abstract

Investigators from Denmark at Aarhus University studied the long-term risk of epilepsy, psychiatric disorders, and mortality among children with recurrent febrile seizures.

Investigators from Denmark at Aarhus University studied the long-term risk of epilepsy, psychiatric disorders, and mortality among children with recurrent febrile seizures. This was a population-based cohort study that used data from the Danish civil registration system. Children born in Denmark between 1977 and 2011 were included. From a cohort of 2,103,232 children, 75,593 children (3.6%) diagnosed with febrile seizures were identified. The risk of febrile seizures peaked at age 16 months, and 90.9% had their first febrile seizure before age three years. History of recurrent febrile seizures appeared to be associated with a risk of epilepsy and psychiatric disorders, but only individuals who later developed epilepsy had an increased risk of mortality. The cumulative risk of recurrent febrile seizures was 22.7% after the first febrile seizure, 35.6% after the second, and 43.5% following the third. The 30-year cumulative incidence of epilepsy increased with the number of hospital admissions for febrile seizures, and it was 2.2% at birth, 6.4% after the first febrile seizure, 10.8% after the second, and 15.8% following the third. The 30-year risk of a psychiatric disorder was 17.2%. After the first febrile seizure, the risk increased to 21.4%, 25% after two or more admissions with febrile seizures, and 29.1% after three or more. Mortality increased with the number of hospital admissions associated with febrile seizures, which was likely explained by a subsequent diagnosis of epilepsy. [1]

COMMENTARY. Febrile seizures are common, affecting 2-5% of children six months to 5 years of age. Several retrospective and prospective studies suggest that 2-7% of children with febrile seizures will later develop epilepsy [2]. Recognized risk factors for epilepsy after febrile seizures are complex features (focality, status epilepticus), abnormal developmental history, and a family history of epilepsy [3]. A prospective study of 560 children with febrile seizures showed that recurrent febrile seizures increased the risk of epilepsy 10-fold, though focality was the highest risk factor [2]. Dreier et al.'s results indicate that recurrent febrile seizures increase the risk for subsequent epilepsy, with the authors showing a cumulative effect based on the number of seizures [1].

Another interesting finding is the association with recurrent febrile seizures and psychiatric disorders; reported previously by the same authors. While the initial analysis in this paper did not detail the types of psychiatric disorders, a subsequent letter to the editor showed the most common psychiatric diagnoses were anxiety, mood, attention-deficit/hyperactivity, and personality disorders [4].

The increased risk of epilepsy in this study is supported by a recent, large retrospective study that found an 18-fold increased incidence of epilepsy in children with recurrent febrile seizure admissions in Taiwan [5]. However, both studies have similar limitations by relying on hospital admissions and lacking classification between simple and complex febrile seizures. Many patients with simple febrile seizures could have been missed in this study, as these children are not likely to require admission.

Nevertheless, the current study adds important prognostic information regarding recurrent febrile seizures and the long-term risk of epilepsy, neuropsychiatric outcomes, and mortality.

## Disclosures

The authors have declared that no competing interests exist.
